# Preliminary comparison of gun homicide deaths for non-probation and probation clients.

**DOI:** 10.23889/ijpds.v7i3.2098

**Published:** 2022-08-25

**Authors:** Kruti Doshi, Keiki Hinami, Jordan Boulger, Huiyuan Zhang, William Trick, Luke Rasmussen

**Affiliations:** 1 Hektoen Institute; 2 Cook County Health; 3 Cook County Adult Probation Department; 4 Northwestern University

## Objectives

To measure a baseline rate of gun homicide deaths for adults on probation in Cook County, Illinois, USA. To compare this rate to the rates for the population of the City of Chicago and for all of Cook County, which includes Chicago and suburban Cook County.

## Approach

In March of 2022, two data contributing partners, Cook County Adult Probation Department (APD) and Cook County Medical Examiner’s Office (CCME), partnered with Cook County Health for salt and crypto generation and the Medical Research Analytics and Informatics Alliance (MRAIA) as the data generator. We used the open source Linkja program (\url{https://linkja.github.io/}) to match adult probation records with gun homicide death records from CCME. For our study period, CY 2018 through 2021, APD submitted a hashed roster of 53,969 unique individuals active on probation, which was matched to 307 records of gun homicide deaths.

## Results

Overall, there were 3,728 gun homicides in the CCME data, 8% (n=307) involved individuals on probation. The average age was 34 years at death, almost 91% were male (n=3,374), 71% Black (n=2,650), and 14% Hispanic (n=536). Among individuals who died from gun violence, compared to those not in the probation data, probation clients tended to be younger (29 vs 33, p<.001) and more likely to be Black (89% vs. 69.5%; p<.001),

We found that the rate of gun homicide deaths per 100,000 probation clients was 569, ~22 times higher than the rate for the City of Chicago in 2020 (rate=25 per 100,000) and ~33 times higher than the rate for Cook County in 2020 (rate=17 per 100,000).

## Conclusion

Privacy-preserving record linkage software enabled a cross-departmental data sharing project that heretofore was not realized due to privacy concerns and existing data use agreements. Our results highlight an excessive risk of homicide among adult probation clients, particularly among young black males.

